# Visualization and Characterization of the Brain Regional Heterogeneity of Astrocyte–Astrocyte Structural Interactions by Using Improved Iontophoresis with Dual-Fluorescent Dyes

**DOI:** 10.3390/brainsci13121644

**Published:** 2023-11-27

**Authors:** Qingran Li, Bin Zhou, Mengchan Su, Ping Liao, Fan Lei, Xin Li, Daqing Liao, Xia Zhang, Ruotian Jiang

**Affiliations:** 1 Department of Anesthesiology, West China Hospital of Sichuan University, Chengdu 610041, China; 2 Laboratory of Anesthesia and Critical Care Medicine, National-Local Joint Engineering Research Center of Translational Medicine of Anesthesiology, West China Hospital, Sichuan University, Chengdu 610041, China; 3 Department of Neurology, West China Hospital of Sichuan University, Chengdu 610041, China

**Keywords:** astrocyte–astrocyte interaction, morphology, territory, iontophoresis

## Abstract

Astrocytes are morphologically intricate cells and actively modulate the function of the brain. Through numerous fine processes, astrocytes come into contact with neurons, blood vessels, and other glia cells. Emerging evidence has shown that astrocytes exhibit brain regional diversity in their morphology, transcriptome, calcium signaling, and functions. However, little is known about the brain regional heterogeneity of astrocyte–astrocyte structural interaction. So far, the visualization and characterization of the morphological features of adjacent astrocytes have been difficult, and as a result, it is still well-accepted that astrocytes in the adult brain share non-overlapped territory. In contrast, employing an approach that combines viral labeling with dual-fluorescent dyes iontophoresis under brightfield and imaging using confocal microscopy allows for the efficient and specific labeling of adjacent astrocytes, enabling a comprehensive visualization of their fine processes and the degree of their territorial overlap. Our study in the hypothalamic regions of the brain revealed a marked spatial overlap among adjacent astrocytes, which differs from the conventional understanding based on more extensively studied regions, like the hippocampus. Additionally, we revealed the heterogeneity of the astrocyte–neuron ratio across brain regions and conducted an assessment of the photostability and labeling efficiency of fluorescent dyes used for labeling adjacent astrocytes. Our study provides new insights for studying the morphological heterogeneity of astrocytes across the central nervous system.

## 1. Introduction

Astrocytes are an abundant type of glia in the central nervous system (CNS). Astrocytes have high complex morphology with the majority of their cell volume consisting of numerous fine processes through which they come into contact with neurons, blood cells, and other types of glia cells. It is well-known that astrocytes are actively involved in various physiological processes of the nervous system, including synapse formation and maintenance, the metabolic support of neurons, and the regulation of blood flow [1,2]. Undoubtedly, the diverse functions of astrocytes in the central nervous system are built upon their intricate morphological features. 

The structural and functional interactions of astrocytes and neurons, especially with synapses, have been well investigated [3]. On the contrary, astrocyte–astrocyte interactions are less well understood. By using the fluorescent dye iontophoresis technique, Bushong et al. showed that adjacent astrocytes in the hippocampal CA1 region possess independent spatial domains with minimal overlap between their branches [4]. From then on, the concept that astrocytes in the adult brain have non-overlapping territory has become widely accepted [5,6,7,8,9], which has been further strengthened by studies on human and drosophila brains [10,11]. However, it is noteworthy that astrocytes in the ferret visual cortex share half of their territory with other astrocytes [12], and the processes of the interdigitation of adjacent astrocytes were also reported in the colliculus in a study using a Brainbow transgenic mice line [13].

It is widely accepted that astrocytes exhibit brain regional heterogeneity in terms of morphology, transcriptome, and function [14,15,16,17,18,19]. However, the brain regional heterogeneity of astrocyte–astrocyte interaction is still awaiting elucidation. There has been an urgent need to develop new methods to visualize and characterize the structural interaction between adjacent astrocytes clearly and efficiently. Previously reported methods of labeling astrocytes with fluorescent dyes (iontophoresis or dye filling) [4,12,20] mostly relied on the identification of astrocyte cell bodies under brightfield microscopy, which is time- and labor-consuming and may be challenging in brain regions with high cell density or neural filaments. And, the drawbacks of using genetically labeled astrocytes with fluorescent proteins, such as Cre-dependent MORF (mononucleotide repeat frameshift) [21] and MADM (mosaic analysis with double markers) [22], include long cycle times, high costs, limited resolution, and low efficiency to label the adjacent astrocytes with different fluorescent proteins. 

To this end, to explore the structural interactions between adjacent astrocytes in broad brain regions, we developed a method that involves iontophoresis with dual-fluorescent dyes under brightfield with the guidance of fluorescent protein introduced by AAV, followed by confocal imaging. This method exhibits a high signal–noise ratio in displaying the fine structure of astrocytes with high efficiency. More importantly, we found that the fine processes of astrocytes in the VMH (ventromedial hypothalamus) invaded the territory of the neighboring ones, forming a high degree of overlap, which is typically not observed in previously studied brain regions, such as the cortex or hippocampus.

## 2. Materials and Methods

### 2.1. Ethics Statement

All experiments involving mice were approved by the Animal Research Committee at the West China Hospital of Sichuan University (protocol 20230220023).

### 2.2. Animals

Six-week-old C57BL/6 mice were purchased from Gempharmatech Co., Ltd. (Nanjing, China) and housed in a temperature- and humidity-controlled room with a 12 h light–dark cycle and provided with ad libitum access to water and food. Only male mice were included in the experiment. Pregnant mice were purchased from Dossy Biotechnology (Chengdu, China), and their newborn pups underwent surgery on the first day after birth (P0). Subsequently, the newborn mice were kept with the maternal mice until 3–4 weeks of age.

### 2.3. AAV Microinjections

AAV5-GfaABC_1_D-EBFP, AAV5-GfaABC_1_D-EGFP, and AAV5-GfaABC_1_D-mCherry were purchased from Taitool Bioscience Co., Ltd. (Shanghai, China). To sparsely label astrocytes in the different brain regions, the newborn mice (P0) were cryo-anesthetized before injection as previously reported [23]. Viral injections were performed by using a stereotaxic apparatus (RWD, Shenzhen, China) to guide the placement of a Hamilton syringe fixed with beveled glass pipettes (Sutter Instrument, Novato, CA, USA, B100-58-10) into the cortex and hypothalamus. The coordinate for the cortex was 1.5 mm anterior to the posterior fontanelle, 1.2 mm lateral to the midline, and 0.6 mm below the surface of the skin, and the coordinate for the hypothalamus was 1.5 mm anterior to the posterior fontanelle, 0.2 mm lateral to the midline, and −3.4 to −3.7 mm below the surface of the skin. To test the labeling efficiency of each fluorescent protein, a total of 1.0 µL of AAV5-GfaABC_1_D-EBFP (2.0 × 10^12^ gc/mL), AAV5-GfaABC_1_D-mCherry (2.0 × 10^12^ gc/mL), or AAV5-GfaABC_1_D-EGFP (2.0 × 10^12^ gc/mL) was slowly injected into both sides of the hemisphere. For combined injection of EBFP and EGFP, a total of 0.5 µL of AAV5-GfaABC_1_D-EBFP (2.0 × 10^11^ gc/mL) and 0.5 µL of AAV5-GfaABC_1_D-EGFP (2.0 × 10^11^ gc/mL) were mixed (1:1 in volume) and injected into the cortex of one hemisphere and the hypothalamus of the other hemisphere of the brain. The glass pipette was left in place for at least 5 min. After injection, the pups were allowed to completely recover on a warming blanket and then returned to the home cage. As for the six-week-old mice, 1.0 µL of AAV5-GfaABC_1_D-EBFP (2.0 × 10^12^ gc/mL) was injected into hippocampal CA1sr (anterior–posterior: −2.10 mm, mediolateral: ±1.45 mm, and dorsal–ventral: −1.47 mm) and 0.8 µL of the virus was injected into the VMH (anterior–posterior: −1.50 mm, mediolateral: ±0.50 mm, and dorsal–ventral: −5.60 mm). Throughout the surgical procedure, the mice were maintained in a stable anesthetized state with 1.2%–1.5% isoflurane and were allowed to recover on a warming blanket after surgery.

### 2.4. Immunohistochemistry (IHC)

The six-week-old male mice were deeply anesthetized with isoflurane and then perfused transcardially with cold phosphate-buffered saline (PBS), followed by 10% neutral formalin. Subsequently, their brains were carefully removed and immersed in 10% formalin at 4 °C overnight. The following day, they were dehydrated in a 30% sucrose solution for 3–4 days. Coronal sections (20 mm) were collected using a cryostat microtome (Leica, Wetzlar, Germany #cm1860) at −20 °C, washed three times in PBS for 10 min, and incubated with 10% normal goat serum (containing 0.5% Triton X-100) for 1 h at 37 °C. Next, the brain sections were incubated with primary antibodies in 5% normal goat serum within 0.05% Triton X-100 overnight at 4 °C. The following primary antibodies were used: mouse anti-NeuN (1:500, Millipore, Burlington, VT, USA, #MAB377), mouse anti-Kv2.1 (1:100, NeuroMab, Davis, CA, USA, #75-014), and rabbit anti-S100β (1:500, Proteintech, Chicago, IL, USA, #15146-1-AP). The following Alexa-conjugated secondary antibodies were used: goat anti-mouse 555 (1:500, Abcam, Cambridge, UK, #ab150114), goat anti-mouse 488 (1:1000, Abcam, Cambridge, UK, #ab150113), and goat anti-rabbit 488 (1:1000, Abcam, Cambridge, UK, #ab150077). After nucleus labeling with DAPI, the coverslips were mounted on slides using anti-fade solution. The confocal images were obtained with a 10× air lens and a 60× oil-immersion lens.

### 2.5. Iontophoresis with Fluorescent Dyes and 3D Reconstruction

The protocols of iontophoresis with fluorescent dyes have been described previously [4,24], with slight adjustments. Mice injected with AAV5-GfaABC_1_D-EBFP before were perfused transcardially with PBS (35–37 °C), followed by 10% neutral formalin (35–37 °C). The dissected brains were post-fixed in PBS diluted 5% neutral formalin overnight at 4 °C. On the following day, 100 mm brain sections containing the target brain region were prepared by using a vibrating microtome (Leica, Wetzlar, Germany, #VT 1200s). The sections were kept in PBS and visualized by using a Nikon A1R+ confocal microscope (Tokyo, Japan) through an infrared camera (DAGE MTI, Michigan City, IN, USA, IR1000) and a 40× water immersed lens. EBFP-positive astrocytes were identified, and a pair of cells with adjacent cell bodies in the same focal plane were selected. Subsequently, 1.5% Lucifer yellow dilithium salt (Lucifer yellow, Merk, Darmstadt, Germany, #67769-47-5) and 2 mM Alexa Fluor 568 Hydrazide (Alexa 568, Invitrogen, Carlsbad, CA, USA, #A10441) were separately iontoporesed [25] into the adjacent astrocytes by using a pipette (Sutter Instrument, Novato, CA, USA, BF100-58-10) with an extremely fine tip and a self-made electrical circuit. Thereafter, the sections were mounted on glass slides by using the anti-fade mounting medium (Vector Laboratories, Burlingame, CA, USA, H-1000) and stored at 4 °C. Clear images of astrocytes were obtained by using a confocal microscope (Nikon A1R+, Tokyo, Japan) equipped with a 60× oil-immersion lens. The Z-step size was 0.25 μm and the Z-intensity correction function was used. Three-dimensional reconstructions were processed offline by using the surface function of Imaris (version 10.0.0, Bitplane, Zurich, Switzerland), as reported previously [26]. The overlapped territory was calculated automatically when the territories of Lucifer yellow- and Alexa 568-positive astrocytes were reconstructed. The percentage of overlapped territory was calculated as *V_Overl__ap_****/***$\frac{1}{2}$ *(V_Lucifer_ + V_Alexa568_)* × 100%, where *V* represents the volume of astrocytic territory.

### 2.6. Photostability of Fluorescent Dyes

As for measuring the photostability of different fluorescent dyes, astrocytes in the formalin-fixed brain slices from WT mice that contained hippocampal CA1sr were selected randomly. And, 1.5% Lucifer yellow, 2 mM Alexa Fluor 488 (Invitrogen, Carlsbad, CA, USA, #A10436), 2 mM Alexa 568, and 5 mM Sulforhodamine 101 (SR101, Sigma-Aldrich, St. Louis, MI, USA, 60311-02-6) were iontophoresed into different cells. Thereafter, time-lapse imaging (0.1 Hz) was conducted using a confocal microscope (Nikon A1R+, Tokyo, Japan) with a 40× water-immersion objective lens (numerical aperture, NA 0.8) for 5 min. Finally, the brain slices were mounted on a glass slide by using the anti-fade mounting medium. And, confocal microscopy was performed by using a 60× oil-immersion lens after 3 days.

### 2.7. Statistical Analyses

Statistical analyses were conducted using GraphPad Prism (version 8.0, San Diego, CA, USA). All data are presented as the mean ± SEM. The Kolmogorov–Smirnov normality test was used to test for normal distribution. Data fitting a parametric distribution were tested for significance using analysis of unpaired Student’s two-tailed *t*-tests; data fitting a non-parametric distribution were tested for significance using two-tailed Mann–Whitney U tests. Data with more than two groups were tested for significance using a one-way ANOVA test followed by post hoc Tukey’s test (parametric data) or Kruskal–Wallis test followed by Dunn’s multiple comparisons test (non-parametric data). Significance was defined as *p* < 0.05.

## 3. Results

### 3.1. Brain Regional Heterogeneity of Cell Density and Astrocyte–Neuron Ratio

Sparse labeling of astrocytes using iontophoresis with fluorescent dyes offers abundant morphological details. However, this method is relatively laborious and time-consuming. Previous studies investigating single and adjacent astrocytic morphology using this method mainly focused on the hippocampus and cortex, two brain regions that are relatively large and abundant with astrocytes. To explore the heterogeneity in astrocyte–astrocyte interactions across the cortical and subcortical brain regions, like the hypothalamus, we first evaluated the astrocyte–neuron ratio in the stratum radiatus layer of hippocampal CA1 (CA1sr), the somatosensory cortex, the thalamus, the striatum, and the ventromedial hypothalamus (VMH).

**Figure 1:**
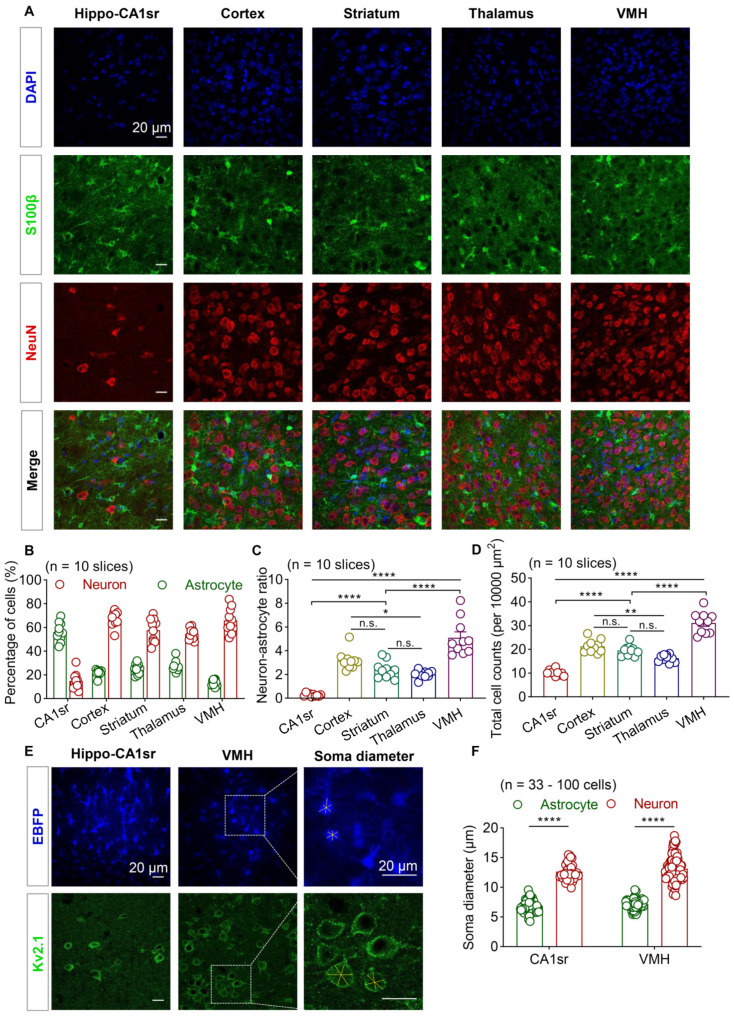
Brain regional heterogeneity of cell density and astrocyte–neuron ratio. (**A**) Representative immunofluorescence images of astrocytes (S100β, green) and neurons (NeuN, red) in the hippocampal CA1sr, cortex, striatum, thalamus, and VMH. Scale bars, 20 µm. (**B**) Quantification of the proportion of astrocytes and neurons within each brain region (n = 10 slices for each brain region). (**C**) Quantification of the relative ratio of neurons to astrocytes (n = 10 slices; one-way ANOVA followed by Tukey’s multiple comparisons test). (**D**) Quantification of total cell counts per 10,000 µm^2^ (n = 10 slices; one-way ANOVA followed by Tukey’s multiple comparisons test). (**E**) Representative images of astrocytes labeled by AAV5-GfaABC_1_D-EBFP and neurons labeled by Kv2.1 in the hippocampal CA1sr and VMH. The measurement method of soma diameter is shown on the right. The yellow dashed lines show the three randomly selected branches of each cell. Scale bars, 20 µm. (**F**) Quantification of astrocyte and neuron soma diameter in the hippocampal CA1sr and VMH (n = 36–168 cells; unpaired *t*-test). * *p* < 0.05, ** *p* < 0.01, **** *p* < 0.0001, n.s, not significant. Data are shown as the mean ± SEM. VMH, ventromedial nucleus of the hypothalamus; Hippo-CA1sr, hippocampus CA1 stratum radiatus; S100β, S100 calcium-binding protein beta; NeuN, neuron nucleus; AAV, adeno-associated virus; EBFP, enhanced blue fluorescent protein; Kv2.1, voltage-gated potassium channel subfamily KQT member 2.1.

We quantified the total cell count in each field of view from the brain slices, in which the astrocytes and neurons were labeled with S100β and NeuN using IHC, respectively (Figure 1A). Of note, S100β is a pan-astrocytic marker that labels most astrocytes in the cortical and subcortical regions [1,27,28,29]. We then calculated the proportion of astrocytes and neurons within each brain region. We found that in the CA1sr, the astrocytes counted for 56.2% and the neurons counted for 14.9% of the total cells (indicated by DAPI), whereas, in other brain regions, such as the cortex, thalamus, striatum, and VMH, neurons were the predominant cell type (54.6–66.9%) and astrocytes only accounted for 13.5–27.6% (Figure 1B). Subsequently, we conducted a comparison of the neuron–astrocyte ratio across the brain regions. The data revealed that the CA1sr exhibited the lowest ratio, while the VMH displayed the highest ratio when compared to the other regions. The ratio of neurons to astrocytes in the striatum did not show statistically significant differences in comparison to the thalamus or cortex. However, the cortex displayed a slightly higher ratio than the thalamus. (Figure 1C). Additionally, there were variations in the total cell density among the different brain regions. The cell density in the VMH was significantly higher than that in the cortex, thalamus, striatum, and CA1sr. Additionally, the cortex demonstrated a higher cell density than the thalamus. (Figure 1D). Therefore, both the overall cell density and the neuron–astrocyte ratio exhibited regional heterogeneity.

The iontophoresis of astrocytes requires the identification of the cell bodies of astrocytes based on their small size using brightfield microscopy. To characterize the size of the cell bodies in the different brain regions, we used the AAV5-GfaABC_1_D-EBFP virus to label astrocytes in the hippocampal CA1sr and VMH and used Kv2.1 (a neuron-specific marker that is discrete and highly restricted to large clusters on the soma and proximal dendrites [30,31]) to label neuronal cell membranes. We randomly selected three branches for each cell and calculated the average value. We observed that both in the CA1sr and VMH, the neuronal cell bodies were significantly larger than the astrocytic cell bodies (Figure 1E,F). Since CA1sr is a dendritic region with a scarcity of neuronal soma and low cell density, but rich in astrocytes, the classical iontophoresis method can work well in this area. However, considering the high cell density and low astrocyte–neuron ratio in the hypothalamus, it may be challenging to identify astrocytes using brightfield microscopy.

### 3.2. Sparse Labeling of the Adjacent Astrocytes by Using AAVs Expressing Fluorescent Proteins Exhibited Low Efficiency

**Figure 2:**
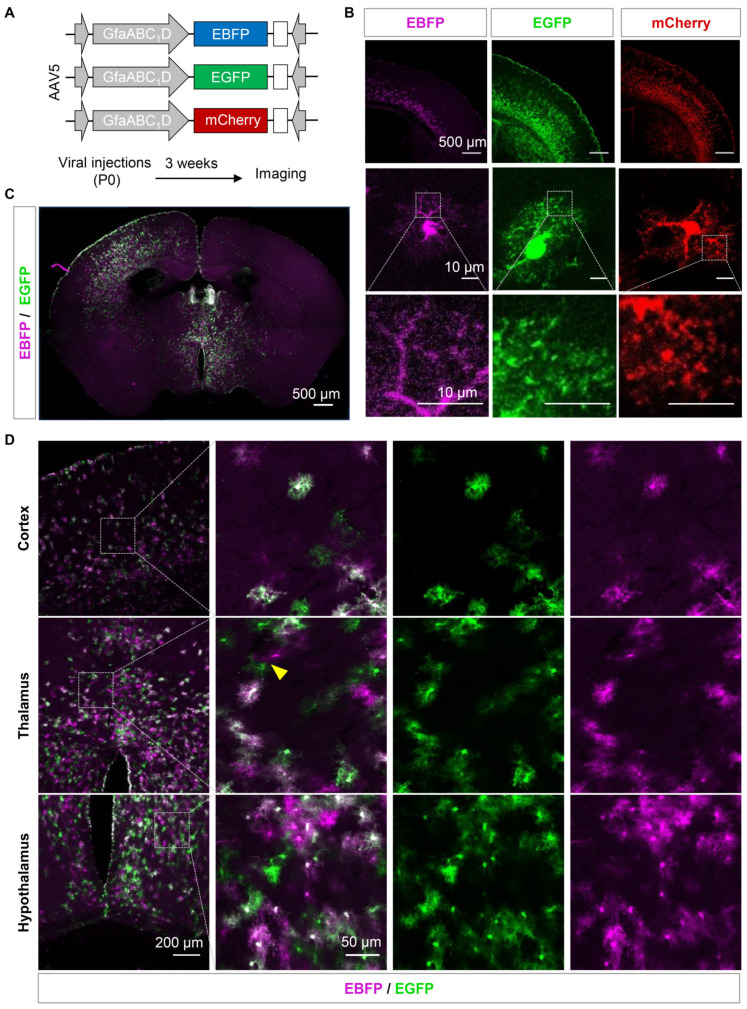
Sparse labeling of the adjacent astrocytes by using AAVs expressing fluorescent proteins exhibited low efficiency. (**A**) The diagram illustrates the vector construction, AAV injection in the neonate mice, and the protocol for brain slice imaging. (**B**) Representative confocal images of the cortex, single astrocyte, and astrocytic fine processes from EBFP-, EGFP-, and mCherry-injected mice. (**C**,**D**) Montage (**C**) and zoomed-in images (**D**) of the EBFP/EGFP-expressing brain. The yellow arrow represents EBFP- and EGFP-positive adjacent astrocyte pairs. Scale bars are shown in the pictures.

Labeling astrocytes by using over-expressing fluorescent proteins is a widely used approach, and the adeno-associated virus (AAV) is a commonly used transfection tool with high efficiency even in non-proliferated cells [32,33,34,35]. Enhanced blue fluorescent protein (EBFP), enhanced green fluorescent protein (EGFP), or mCherry was expressed in the cortex of the adult mice by using AAV5 and the astrocyte-specific promoter GfaABC_1_D. Sparse labeling was achieved through the injection of AAVs into the cortex of the newborn (P0) mice, as described previously [36]. Three weeks later, fluorescent images of virus-labeled astrocytes were obtained by using confocal microscopy (Figure 2A). EGFP and mCherry were more widely expressed than EBFP in the cortex of the mice, and sparsely labeled astrocytes were also detected (Figure 2B). Among the three fluorescent proteins, EGFP provided the best representation of the complete cellular morphology and fine processes of astrocytes. EBFP exhibited weak fluorescence and a low signal-to-noise ratio, making it difficult to visualize the fine processes of astrocytes. On the other hand, mCherry tended to form aggregates and did not effectively display the intricate branching patterns (Figure 2B). In addition, tdTomato is also a commonly used red fluorescent protein, but its susceptibility to photobleaching under confocal imaging may make it unsuitable for our experiment [37].

Subsequently, we attempted to achieve sparse labeling by co-injecting two low-concentration fluorescent viruses, EGFP and EBFP, into the cortex and hypothalamus of the P0 mice with the hope of labeling adjacent astrocytes with two different colors. After three weeks, scattered EBFP- and EGFP-positive astrocytes were observed in the cortex, hypothalamus, and subcortical regions (Figure 2C). Most cells showed a simultaneous expression of EBFP and EGFP, while only a small number of astrocytes expressed only one of the two fluorescent proteins. Additionally, only a few pairs of adjacent astrocytes were detected, expressing EBFP and EGFP separately and in proximity. In a field of view measuring 6.4 × 10^4^ µm^2^, only 1–2 pairs of astrocytes successfully labeled with both colors and located close enough to each other were found (Figure 2D). The labeling efficiency failed to improve when we increased the viral tilter and volume or changed the ordination of injections. We conducted a simple statistical analysis and found that only approximately 22.33% of the astrocytes were individually labeled with a single fluorescent protein. Moreover, most adjacent astrocytes were infected with the same color, making it difficult to distinguish the boundaries of their respective territories, and different color pairs were often widely separated from each other. This indicates that the efficiency of labeling adjacent astrocytes using this method is low, especially in smaller brain regions, such as the hypothalamus.

### 3.3. Photostability and Labeling Efficiency of Four Types of Fluorescent Dyes

**Figure 3:**
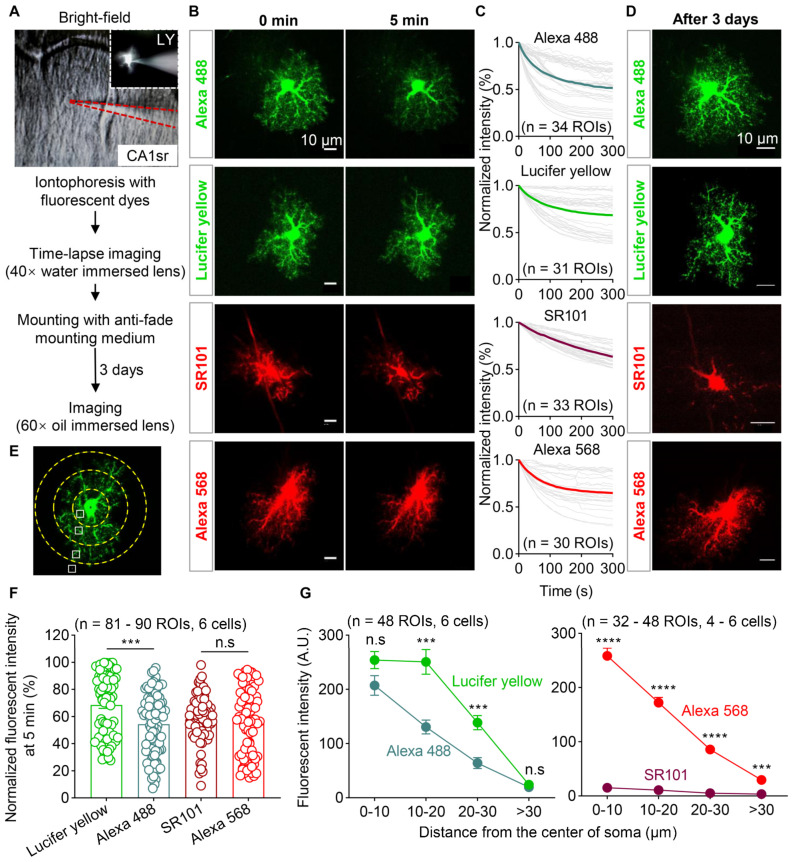
Photostability and labeling efficiency of four types of fluorescent dyes. (**A**) Schematic diagram of iontophoresis with fluorescent dyes conducted in the CA1sr of WT mice under the brightfield and imaging protocol. Upper right image showed the individual astrocyte filled with Lucifer yellow. The area enclosed by the red dashed line indicates the pipette. (**B**) Representative images of astrocytes filled by 2 mM Alexa 488, 1.5% Lucifer yellow, 5 mM SR101, and 2 mM Alexa 568 at the beginning and 5 min after time-lapsing imaging. Scale bars, 10 µm. (**C**) Normalized fluorescence intensity decay over time of the 4 fluorescent dyes (n = 30–34 ROIs). (**D**) Fluorescent images of astrocytes labeled by 4 fluorescent dyes after 3 days of storage at 4 °C. Scale bars, 10 µm. (**E**) The diagram illustrates the location of ROIs selected for fluorescence intensity measurement in astrocytes. (**F**) Quantification of normalized fluorescence intensity of astrocytic processes labeled by Lucifer yellow, Alexa 488, SR101, and Alexa 568 at 5 min (n = 81–90 ROIs from 6 cells; Kruskal–Wallis test followed by Dunn’s multiple comparisons test). (**G**) Quantification of the fluorescence intensity of astrocytic processes labeled with Lucifer yellow and Alexa 488 (left, n = 48 ROIs from 6 cells; two-way ANOVA followed by Sidak’s multiple comparisons test), as well as SR101 and Alexa 568 (right, n = 32–48 ROIs from 4 to 6 cells; two-way ANOVA followed by Sidak’s multiple comparisons test) after 3 days of storage at 4 °C. *** *p* < 0.001; **** *p* < 0.0001, n.s, not significant. Data are shown as the mean ± SEM. LY, Lucifer yellow; ROI, region of interest.

To overcome the low efficiency of labeling adjacent astrocytes with AAVs, we tried iontophoresis with fluorescent dyes, which should have also provided better resolution for morphological assessment. We started this process by evaluating the photon stability, brightness, and degree of leakage for the most used fluorescent dyes. Light-fixed brain slices from wild-type mice containing the hippocampal CA1sr were iontophoresed with Lucifer yellow, Alexa 488, Alexa 568 (hydrazide), or SR101 through a pipette with a fine tip. The astrocytes were easily identified in hippocampal CA1sr, where the cell density was relatively low and astrocytes counted for the majority of cells (Figure 1A–D), under the brightfield by their small, round, or oval cell bodies. Immediately after iontophoresis, time-lapse imaging (10 s intervals and lasting for 5 min) was performed by using the confocal microscope equipped with a 40× water-immersion lens. Thereafter, the brain slices were mounted on glass slides by using the anti-fade mounting medium. And, confocal microscopy was performed by using a 60× oil-immersion lens after 3 days (Figure 3A). The acute decay of fluorescent dyes was detected by analyzing the fluorescent intensity of labeled astrocytes. The fluorescence intensity of Lucifer yellow, Alexa 488, and Alexa 568 exhibited a curve-shaped decay (exponential attenuation), while SR101 showed a nearly linear decay trend (Figure 3B,C,F). Additionally, the fluorescence intensity of Alexa 488 exhibited a high degree of variability between cells, and many cells showed only 20% of the initial fluorescence intensity in certain regions of interest (ROI) after continuous 5-min imaging. In that Alexa 488 was a photostable fluorescent dye [38,39]; our data indicated that it was easy for Alexa 488 to leak from the labeled cells.

The ability of dyes to effectively visualize the fine processes of astrocytes is also crucial. Therefore, we captured images of the iontophoresed cells under a 60× oil-immersion lens to test the fluorescence intensity of astrocytic processes at different levels after 3 days of storage (Figure 3D). Using the approximate center of the astrocytic soma as the origin, we drew concentric circles with radii of 10, 20, and 30 µm. Four ROIs, located in the first circle and edge of the soma (away from the main branches), between the first and second circles, between the second and third circles, or near the third circle, were selected for each astrocyte. We measured the fluorescence intensity of the branches in these four ROIs with the four fluorescent dyes (Figure 3E). Compared to Alexa 488, Lucifer yellow demonstrated better labeling efficiency within the range of 10–30 µm from the soma. As for Alexa 568 and SR101, the fluorescence intensity of Alexa 568 was significantly higher than that of the cells labeled with SR101 in all ROIs. Several astrocytes labeled with SR101 even exhibited complete fluorescence quenching, making imaging highly difficult (Figure 3G). These data indicate that Lucifer yellow and Alexa 568 are more suitable than Alexa 488 or SR101 for labeling astrocytes.

### 3.4. Astrocytes Showed Overlapped Territories in the VMH

**Figure 4:**
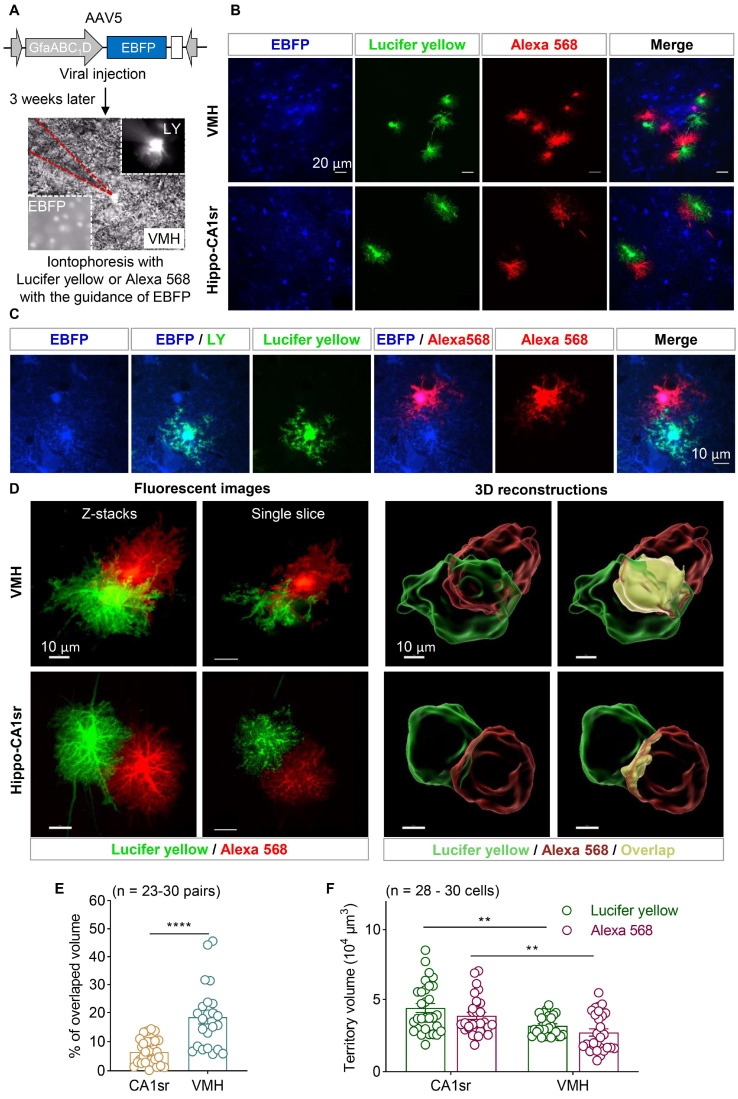
Astrocytes showed overlapped territories in the VMH. (**A**) The diagram illustrated AAV injection of the 6-week-old mice and the iontophoresis protocol. Brightfield image of VMH was displayed. EBFP+ cells were easily distinguished under widefield fluorescence microscopy (lower left), and astrocytes filled with LY exhibited strong fluorescent signals (upper right). The area enclosed by the red dashed line indicates the pipette. (**B**) Representative images of astrocytes labeled by EBFP, Lucifer yellow, and Alexa 568 in the VMH and hippocampal CA1sr. Scale bars, 20 µm. (**C**) A pair of EBFP+ adjacent astrocytes were iontoporesed using Lucifer yellow and Alexa 568, respectively. Scale bar, 10 µm. (**D**) Confocal fluorescent images and 3D reconstructions of adjacent astrocytes in the VMH and hippocampal CA1sr. Scale bars, 10 µm. (**E**) Quantitative percentage of the overlapped volume between the adjacent astrocytes in the VMH and hippocampal CA1sr (n = 23–30 pairs; unpaired *t*-test). (**F**) Quantification of astrocytic territory volume labeled by Lucifer yellow and Alexa 568 in the VMH and hippocampal CA1sr (n = 28–30 cells; two-way ANOVA followed by Sidak’s multiple comparisons test). ** *p* < 0.01; **** *p* < 0.0001. Data are shown as the mean ± SEM.

Notably, the VMH exhibits high cell density and a small proportion of astrocytes (Figure 1). To label the adjacent astrocytes more effectively, AAV5-GfaABC_1_D-EBFP was injected into the VMH of six-week-old mice. Three weeks later, the mice were sacrificed and fixed with formalin and their brain slices were obtained. The astrocyte soma was easily recognized under a brightfield microscope with the guidance of EBFP. The adjacent astrocyte pairs were selected if the distance between their somata was less than 40 µm. Thereafter, Lucifer yellow and Alexa 568 were iontophoresed into each astrocyte, respectively (Figure 4A,C). Astrocytes in the hippocampal CA1sr were also labeled as the control (Figure 4B). Confocal microscopic images showed that a clear boundary existed between the adjacent astrocytes in the hippocampal CA1sr; on the contrary, the Lucifer yellow- and Alexa 568-positive fine processes of astrocytes invaded the territory of each other in the VMH. And, in some cases, the Lucifer yellow-positive fine processes of astrocytes even extended near the soma of Alexa 568-positive neighboring astrocytes (Figure 4D). To quantify the degree of the overlapped territory of astrocytes in the two brain regions, the territories of labeled astrocytes were three-dimensionally reconstructed by using Imaris software. We found that the overlapped territory of astrocytes was only about 6.5% in the hippocampal CA1sr, which was consistent with a previous study. However, the overlapped territory of astrocytes in the VMH was about 18.5%, which was remarkably larger than that in the hippocampal CA1sr (Figure 4E). Furthermore, we evaluated the total territory volume of individual astrocytes in the CA1sr and the VMH. We found that astrocytes in the CA1sr generally had a larger territory volume compared to those in the VMH, but there was no significant difference in the territory volume represented by the two fluorescent dyes (Figure 4F). Together, our data show that dual-fluorescent-dye-iontophoresis with the guidance of fluorescent proteins introduced by AAV is an effective method to label adjacent astrocytes in the brain, especially in high-cell-density brain regions. Importantly, we identified that adjacent astrocytes in the hypothalamus exhibit much larger overlapped territories than those in the hippocampus.

## 4. Discussion

In this current study, we developed a new method that involves dual-fluorescent-dye-iontophoresis with the guidance of fluorescent proteins introduced by AAV for studying the morphological features of adjacent astrocytes. Using this new method, we discovered that mature astrocytes in mice may have highly overlapped territories. Importantly, this method can be applied to study astrocytes in any brain region or even in other species. The relatively smaller size of astrocyte cell bodies compared to neurons, as observed through brightfield microscopy in the thalamus and hypothalamus, along with the low astrocyte–neuron ratio, makes the classical iontophoresis method highly difficult to perform in these regions. Moreover, a microinjection of fluorescent proteins expressing AAVs into the brains of newborn mice showed very low efficiency in labeling adjacent astrocytes. To solve this, we tested and found Lucifer yellow and Alexa 568 (hybridize) to be the best fluorescent dye pairs with high photostability, and they were excellent for illustrating the fine processes of the astrocytes. Last, we labeled the adjacent astrocytes in the VMH and hippocampal CA1sr by using iontophoresis with Lucifer yellow and Alexa 568 with the guidance of EBFP and showed that adjacent astrocytes in the VMH but not in the hippocampal CA1sr have overlapped territories, suggesting that astrocyte–astrocyte interaction also exhibits brain regional heterogeneity.

López-Hidalgo M et al. [12] employed fluorescent dye filling with the guidance of SR101 to investigate astrocyte–astrocyte interaction in the cortex of ferrets. This method allows for the observation of astrocyte morphology in vivo. However, one limitation is the inability to image deep brain regions, such as the thalamus and hypothalamus. Moreover, leakage of fluorescent dye from the filled cells and light scattering in the non-transparent brain reduced the signal-to-noise ratio of the images. This method can also be applied to acute brain slices [20]. However, conducting this experiment on brain slices in situ may subject them to factors such as hypoxia, pH changes, and osmotic pressure, leading to morphological alterations in astrocytes that differ from in vivo conditions. Furthermore, on acute brain slices, due to the preserved cellular activity and intact gap junction functionality between astrocytes, dyes can easily leak and diffuse around the cells when loaded into individual astrocytes. As a result, the complete morphology of astrocytes is not well displayed. If dye loading is performed on acute brain slices, it is necessary to add gap junction blockers. In contrast, our method is based on fixed brain slices, which preserved the physiological cellular morphology observed in live mice. While the permeability of the cell membrane and the functionality of gap junctions were disrupted, it minimized dye leakage to a great extent and ensured the clear visualization of astrocyte fine processes.

Mosaic analysis with double markers (MADM) was introduced by Zong in 2005 [22]. MADM is a genetic engineering method that utilizes gene recombination and selective labeling to study the function and interactions of specific genes in cell populations. By employing the Cre-LoxP system and the targeted insertion of gene cassettes, MADM enables the complementary expression of two fluorescent marker genes in specific cell types, thereby creating a double-labeled cell population. This approach divides cells into four subgroups with distinct marker combinations, allowing researchers to precisely locate and analyze cells. However, MADM has several limitations. It requires the targeted insertion of gene cassettes into specific genomic loci, which necessitates the presence of suitable insertion sites in the target gene. Moreover, MADM relies on the stability of gene cassettes to ensure the proper complementation of the double markers. Some gene cassettes may exhibit high variability and instability, rendering the MADM system unreliable. Furthermore, the complexity of the experimental design, as well as the high costs in terms of time and animal resources, also restrict the application of MADM in labeling neighboring astrocytes. 

Abdeladim et al. [40] introduced a technique called chromatic multi-photon serial (ChroMS) microscopy, which combines multi-photon imaging and multi-color fluorescent labeling. In their study, ChroMS was utilized to investigate the morphology and connections of astrocytes in the mouse cerebral cortex. They achieved multi-color labeling of neighboring astrocytes and confirmed the non-overlapping tiling pattern of the astrocytes in the hippocampus, consistent with previous reports. Clavreul [41] combined ChroMS with the MAGIC Markers (MM) combinatorial labeling strategy to achieve multi-clonal lineage tracing in the mouse cerebral cortex and revealed the plasticity of cortical astrocytes during development. This series of methods enables a comprehensive demonstration of the intricate astrocyte distribution ecology in shallow brain regions, revealing the plasticity during astrocyte development. However, its equipment requirements, substantial data volume, and complex experimental design may limit its broader application, and its effectiveness in deep brain regions has not been validated.

A variety of fluorescent dyes have been utilized for the sparse labeling of astrocytes, including Lucifer yellow, Alexa 568, and Alexa 488. In our study, we demonstrated that Lucifer yellow and Alexa 568 exhibited superior performance compared to Alexa 488 and SR 101. We speculated that the strong polarity of Lucifer yellow and Alexa 568 make it difficult for them to leak from the inotophoresed astrocytes. Even though the fluorescent intensity of EBFP was relatively lower than that of EGFP, making it challenging to visualize the fine processes of the astrocytes, it still provided sufficient brightness to visualize the cell bodies. Consequently, with the aid of EBFP, astrocytes can be easily identified from other cells under brightfield fluorescence microscopy, even in brain regions with high cell density.

Neighboring astrocytes in the VMH exhibit high-level territory overlap. It remains possible that this feature may exist in other hypothalamic regions. The physiological implication of this interesting feature remains unknown. We speculate that the high-level territory overlap may suggest a high level of astrocyte–astrocyte commutations, including Ca^2+^ signaling and possibly energy substrates shuttling between adjacent astrocytes. The molecular mechanism underlying the formation of this territory overlap also requires further study. Analysis of astrocyte intrinsic transcriptional profiles and brain region-specific neuron-to-astrocyte signaling may be necessary. Lastly and importantly, understanding how these astrocyte–astrocyte interactions are altered in disease conditions, such as neurodegenerative diseases or psychiatric disorders, could provide valuable insights into the underlying mechanisms and potentially identify therapeutic targets.

## 5. Conclusions

We achieved adjacent astrocyte labeling using dual-fluorescent-dye-iontophoresis, with the guidance of EBFP introduced by AAV, which could be applied to multiple brain regions or even other species.

After testing several fluorescent dyes, we confirmed that Lucifer yellow and Alexa 568 (hybridize) were the optimal pair due to their high photostability and excellent ability to visualize the fine processes of the astrocytes.

Moreover, we observed significantly higher overlapped territories in the VMH compared to the previously studied hippocampal CA1sr, revealing a brain regional heterogeneity in astrocyte–astrocyte structural interactions.

## Data Availability

All data included in this study are available from the corresponding author upon reasonable request.
